# Brown adipose tissue thermogenesis among a small sample of reindeer herders from sub-Arctic Finland

**DOI:** 10.1186/s40101-022-00290-4

**Published:** 2022-04-20

**Authors:** Cara Ocobock, Päivi Soppela, Minna Turunen, Ville Stenbäck, Karl-Heinz Herzig

**Affiliations:** 1 Department of Anthropology, University of Notre Dame, Corbett Family Hall 296, Notre Dame, IN 46556 USA; 2 Eck Institute for Global Health, Institute for Educational Initiatives, University of Notre Dame, Corbett Family Hall 296, Notre Dame, IN 46556 USA; 3 University of Lapland, Arctic Centre, Rovaniemi, Finland; 4 Research Unit of Biomedicine, Medical Research Center, Faculty of Medicine, University of Oulu, and Oulu University Hospital, Oulu, Finland; 5 Biocenter Oulu, Oulu, Finland; 6 Department of Pediatric Gastroenterology and Metabolism, Poznan University of Medical Sciences, Poznan, Poland

**Keywords:** Brown adipose tissue, Cold stress, Non-shivering thermogenesis, Metabolism, Respiratory quotient

## Abstract

**Introduction:**

Interest in human physiological responses to cold stress have seen a resurgence in recent years with a focus on brown adipose tissue (BAT), a mitochondria dense fat specialized for heat production. However, a majority of the work examining BAT has been conducted among temperate climate populations.

**Methods:**

To expand our understanding of BAT thermogenesis in a cold climate population, we measured, using indirect calorimetry and thermal imaging, metabolic rate and body surface temperatures of BAT-positive and BAT-negative regions at room temperature, and mild cold exposure of resting participants from a small sample of reindeer herders (*N* = 22, 6 females) from sub-Arctic Finland.

**Results:**

We found that most herders experienced a significant mean 8.7% increase in metabolic rates, preferentially metabolized fatty acids, and maintained relatively warmer body surface temperatures at the supraclavicular region (known BAT location) compared to the sternum, which has no associated BAT. These results indicate that the herders in this sample exhibit active BAT thermogenesis in response to mild cold exposure.

**Conclusions:**

This study adds to the rapidly growing body of work looking at the physiological and thermoregulatory significance of BAT and the important role it may play among cold stressed populations.

**Supplementary Information:**

The online version contains supplementary material available at 10.1186/s40101-022-00290-4.

## Introduction

Much of biological anthropology in the past 50 years has focused on human health and disease, with less focus on how physical environments influence human biology. However, with the impacts of climate change growing ever more dire, scholars have begun to better integrate human biology, health, disease, and the physical environment into their research [1–4]. Though in many places climate change will lead to warming, other localities will experience an increase in extreme cold weather events, while drastically different day- and night-time temperatures will still exist elsewhere [5, 6]. As such, cold will always be a stress with which humans will need to cope [7]. Though humans are a hot climate originating species [8], a variety of adaptations have evolved in order to survive and thrive in the relatively recent stressor—extreme cold [7]. One such adaptation is brown adipose tissue (BAT), the main driver of non-shivering thermogenesis. Here, we present the results of BAT activity measurements conducted among a small sample of reindeer herders living in sub-Arctic Finland.

We are physiologically limited in our ability to maintain our core body temperature in the face of cold stress; thus, we also employ behavioral and cultural mechanisms that enable us to survive and thrive in extreme cold environments. Much of the work revolving around these mechanisms focuses on vascular and metabolic responses to cold stress. Vascular responses, which work to reduce heat loss, include peripheral vasoconstriction and countercurrent heat exchange [7, 9, 10]. Metabolic mechanisms, which increase body heat production, include shivering thermogenesis [9], increased resting metabolic rate (RMR, kcal/day) [11–18], and non-shivering thermogenesis via brown adipose tissue [19–23]. Shivering, produced by small muscle contractions in response to cold stress, does not substantially increase body heat production nor persist for a long period of time [24]. RMR, the metabolic cost of maintenance, among cold climate populations is typically higher than expected based on predictive equations and higher relative to temperate climate populations [11, 12, 14, 16]. This is thought to be driven by high levels of thyroid hormone [11, 13, 14, 25], but there is also a high degree of interindividual RMR variation [17]. Among the herders in the present study, after controlling for body and fat-free mass, females had significantly higher RMRs than predictive equations and significantly higher RMRs than males. Males, however, showed no distinct pattern relative to predictive equations [18], only partially matching the pattern seen among other cold climate populations.

Brown adipose tissue is a mitochondria dense tissue that interrupts the electron transport chain via mitochondrial uncoupling protein-1, resulting exclusively in heat rather than adenosine triphosphate production [19, 21, 26, 27]. BAT presence has been well known among hibernating and newborn mammals and human infants, but is, as we now know, also present in healthy adults in cold [19, 28] and temperate climate populations [20, 23, 26, 27, 29, 30]. BAT deposits (~ 100 g) are most prominent in supraclavicular and paracervical regions as well as along major deep blood vessels and around numerous internal organs [31]. These deposits consist of brown and beige adipose tissue, with beige adipose tissue having a reduced thermogenic capacity and is also found among white adipose tissue deposits [32]. Beige adipose tissue is not discussed nor analyzed here.

Those with more BAT thermogenesis typically experience a greater increase in metabolic rate, warmer body surface temperatures over BAT positive regions, and higher glucose utilization in response to mild cold stress (~ 10-15 °C ) [33–35]. This thermogenesis is induced by the sympathetic nervous system and thyroid hormones. Among individuals with active BAT, there is little decline or even an increase in body surface temperatures at the supraclavicular (known BAT location) region during mild cold exposure, whereas individuals without active BAT show significant surface temperature declines in this region [19, 20, 36]. While surface temperature patterns are typically consistent, there is currently no consensus on BAT substrate utilization and some have suggested a higher glucose utilization during BAT activity [19, 36, 37], while others found greater fatty acid utilization. Chondronikola et al. [36] found that the increase in metabolic rate associated with BAT was 70% fueled by free fatty acids and 30% by glucose, while others found an increase in low-density lipoprotein and high-density lipoprotein (HDL) cholesterol levels [38], indicating a high degree of variation in BAT substrate utilization [39, 40]. The amount of BAT is inversely associated with body-mass index, body adiposity, and age [29, 41].

Our understanding of inter- and intra-populational variation, morphological correlates, metabolic responses, genetic indicators, and potential therapeutic benefits of BAT are still limited. Among the Yakut of Siberia, males experienced a decrease in metabolic rate during mild cooling, though no decline in surface temperatures, while females did not [19]. Potential explanations for this decline in metabolic rate may be habituation, increased vasoconstriction, or the Q_10_ effect, which is slowing of enzymatic activity in muscle due to cold exposure though the true cause is still unknown [19, 42, 43]. In the present study, we assessed BAT activity by measuring metabolic rate, substrate utilization, and surface temperatures during acute, mild cold exposure among a small sample of reindeer herders from sub-Arctic Finland. Though our sample size is small, we expected the reindeer herders in this study to have active BAT in response to mild cold exposure, as has been documented among another cold climate population. This work expands our current understanding of populational variation in BAT activity as a response to cold stress.

## Methods and participant population

### Participants

The results of this work are one part of a larger project, *The metabolic cost of living among reindeer herders of sub-Arctic Finland*, that took place during October of 2018 during the annual herd roundup and in January 2019. The BAT measurements discussed here took place in January of 2019. Reindeer herders (*N* = 22, 6 females, and 16 males) from herding districts within 180 km of Rovaniemi, Finland (66.5° N, 25.7° E) participated in this study. The herding districts included Palojärvi, Niemelä, Narkaus, Pyhä-Kallio, Oivanki, Isosydänmaa, and Poikajärvi; for a map, please refer to Ocobock et al. [18]. The skewed sex ratio in this study reflects the actual sex ratio among reindeer herders in Finland, among whom 30% are female [44]. Herders were recruited through existing contacts as well as advertising by the Reindeer Herders Association, and interested herders were contacted by the research team. This resulted in a fairly large age range (20–64 years) among the participating herders. For half of the participating herders, reindeer herding was their primary occupation. For the other half, herders supplemented their income with tourism, meat processing, land measurement, research, and construction. In Rovaniemi, the mean temperature in January of 2019, when BAT measurements were conducted, was − 16.4 °C (2.5 °F) [45].

The Arctic Centre of the University of Lapland in Rovaniemi served as the operation base for the larger study and was the location for much of the data collection. However, when herders were unable to come to the Arctic Centre, measurements were conducted at herders’ homes, cabins within the herding district, or hotel rooms near the herding district of interest. At the time of participation, all herders were active members of their herding district and self-reported being healthy at the time of measurement. All participating female herders were not pregnant or lactating; however, information on menstrual cycle phase was not documented during this study.

This study was conducted with Institutional Review Board approval from the University at Albany (17-E-165) and with the approval of the Ostrobothnian Health Care District from the University of Oulu, Finland (EETTMK: 4/2018). Participants were provided with an oral introduction and written information sheet about the study, and informed written consent (written in Finnish) was obtained from all participants [46].

### Anthropometrics

Height and weight were measured following standard procedure using a portable stadiometer (Seca Corporation, Hanover, MD) and an electronic scale (AccuWeight, New York, NY), respectively. Height was recorded to the nearest 1 mm, and weight was recorded to the nearest 0.1 kg. Body composition was measured using bioelectrical impedance (RJL systems, Clinton, MI). Participants were in a supine position, with arms and legs abducted from the body, and small electrodes were placed on the right side of the body at the dorsum of the wrist, middle finger, ankle, and middle toe. Reactance and resistance were recorded and the NHANES-III equations were used to determine fat-free mass (FFM) and body fat percentage [47]. All participants were asked to refrain from consuming alcohol in the 24 h before measurement, and they arrived to the data collection period 12 h fasted. Total cholesterol, glucose, and HDL cholesterol levels were measured immediately after the anthropometric measurements and before the metabolic measurements. Finger prick whole blood samples were analyzed using the CardioChek PA (Polymer Technology Systems, Indianapolis, IN) point of care devise with glucose-cholesterol test strips.

### Brown adipose tissue and respiratory quotient

BAT thermogenesis was non-invasively inferred through the simultaneous measuring of metabolic rate and surface temperatures of herders, at an anticipated BAT and non-BAT region, during mild cold exposure following Levy [27, 48]. BAT thermogenesis is indicated by an increase in metabolic rate and higher surface temperatures at a BAT region, the supraclavicular region (*T*_SC_) compared to a non-BAT region at least among humans, the sternum (*T*_ST_). In order to assess the impact BAT thermogenesis has on these two parameters, metabolic rate and surface temperatures were first measured in resting subjects at room temperature (20–27 °C) and then measured again at mild cold exposure (15–18 °C), which lasted for 30 min. To expose participants to mild cold, they wore a 3-piece liquid cooling garment (BCS4, Allen Vanguard; Ontario, Canada) fitted with internal tubing through which cold water flowed. For the room temperature condition, no water flowed through the suit.

Prior to the start of data collection, participants put on the 3-piece cooling suits. Participants were then asked to rest in the supine position at room temperature for 30 min prior to the start of measurements allowing them to come to a more complete rest, familiarize themselves with the environment, and minimize possible anxiety associated with testing. Room temperature metabolic rate (MR_RT_, kcal/day) and respiratory quotient (RQ_RT_, VCO_2_/VO_2_) were measured using a K5 portable indirect calorimetry unit (Cosmed, Chicago, IL) for 30 min with the last 10 min of data used for analysis. This system utilizes a facemask with bi-lateral, unidirectional valves (allowing inspiration but not expiration), which is preferable to a metabolic hood as the hood interferes with thermal imaging of the key BAT locations. The K5 unit measures breath-by-breath O_2_ consumption and CO_2_ production. MR_RT_ and RQ_RT_ values were calculated and recorded using the Cosmed Omnia software. In general, a RQ value closer to 1.0 indicates carbohydrates utilization, while a RQ closer to 0.70 signifies that fat is the primary metabolic substrate.

During the 30-min MR_RT_, thermal images of a BAT region and a non-BAT region were taken every 5 min using an E75 thermal imaging camera (FLIR, Wilsonville, OR) held 30 cm from the area of interest with an emissivity of 0.98. The supraclavicular region (a known BAT location) was defined medially by the sternocleidomastoid muscle, laterally by greater tubercle of the humerus, and inferiorly by the clavicle. The sternum served as a control as BAT is not found here. The images were uploaded to the FLIR Tools+ software, and maximum temperature of the supraclavicular and sternal regions were recorded.

Once the 30-min room temperature exposure was complete, cold water (7–10 °C) was pumped into the cooling suit which takes roughly 2–3 min to completely fill resulting in exposure temperatures of approximately 15–18 °C. The cold exposure similarly lasted 30-min, and cold metabolic rate (MR_C_), RQ (RQ_C_), and surface temperatures were all measured exactly the same as described above for the room temperature exposure. Participants were monitored the entire time for potential shivering, and participants were instructed to alert the researcher if shivering started. Shivering was only observed in two participants, the time of shivering was noted so that data could be removed from analysis, and warm water was added to the cooling suit to keep the participants at mild cold exposure. These shivering episodes lasted no longer than 60 s.

### Statistical analyses

We conducted all statistical analyses using SPSS version 26 (IBM, Armonk, NY), and we produced graphical figures using R Studio (version 2021.09.0). Data were normally distributed (*p* > 0.06 in all cases). One-way ANOVAs were used to compare each age, height, weight, sum of skin folds, percent body fat percentage, FFM, and biomarkers. We used paired Student’s *t*-tests to determine if *T*_SC_, *T*_ST_, metabolic rates, and RQ were significantly different between room temperature and mild cold exposure within each individual. We calculated the difference (∆) and percent change for metabolic rate, RQ, *T*_SC_, *T*_ST_, and the difference between cold exposure surface temperatures for the supraclavicular region (SC_C_) and sternal region (ST_C_). We then performed multiple linear regression analysis to determine if body weight, fat free mass, body fat percentage, sex, age, and biomarkers (glucose, total cholesterol, and HDL cholesterol) were predictors for MR_C_, ∆MR, ∆RQ, ∆T_SC_, ∆T_ST_, and SC_C_–ST_C_. Tables display individual participant results, and pooled results are presented as the mean ± the standard deviation. Multivariate regression tables provide the adjusted *R*^2^, F statistics, *p* values, and *β* coefficient. We considered results significant at the *p* ≤ 0.05 level.

## Results

### Participant anthropometrics

Descriptive statistics and a summary of the means ± the standard deviation for anthropometric, demographic, and biomarker data can be found in Table 1. For the overall sample, age ranged from 20 to 64 years, height ranged from 154.8 to 194.6 cm, body mass ranged from 46.7 to 129.6 kg, FFM ranged from 34.2 to 86.0 kg, and body fat percentage ranged from 20.4 to 40.5%. Females were significantly younger (20–37 years) than males 36–64 years (*F* = 26.557, *p* < 0.01). Among females, age ranged from 20 to 37 years, height ranged from 154.8 to 163.0 cm, body mass ranged from 46.7 to 78.9 kg, FFM ranged from 32.2 to 46.9 kg, and body fat percentage ranged from 26.8 to 40.5%. Among males, age ranged from 36 to 64 years, height ranged from 168.0 to 194.6 cm, body mass ranged from 62.9 to 129.6 kg, FFM ranged from 48.2 to 86.0 kg, and body fat percentage ranged from 20.4 to 36.8%. Females were significantly shorter (*F* = 33.168, *p* < 0.01) and had significantly lower body mass (*F* = 11.282, *p* < 0.01) and FFM (*F* = 31.043, *p* < 0.01) relative to males. Females had significantly more body fat than males (*F* = 16.370, *p* < 0.01). There was no significant difference between females and males for any of the biomarker measures (*p* > 0.2 in all cases).

**Table 1:** Anthropometric and blood biomarker (glucose, total cholesterol, and HDL cholesterol) data for each participant as well as the sample means. Age and sex data have been removed to protect participant identity

*Participant*	*Mass (kg)*	*Height (cm)*	*Fat free mass (kg)*	*Body fat percentage*	*Glucose (mmol/L)*	*Total cholesterol (mmol/L)*	*HDL cholesterol (mmol/L)*
1	81.3	168.0	60.1	26.3	5.6	4.6	1.5
2	95.5	174.6	67.2	29.6	5.2	4.9	1.4
4	78.9	163.0	46.9	40.5	6.7	5.5	1.1
5	129.6	194.6	86.0	33.6	5.9	4.0	1.5
6	96.8	181.0	75.1	22.4	4.6	4.8	1.6
7	74.0	178.8	50.7	20.8	4.7	7.9	2.2
8	82.2	178.5	61.3	25.5	5.6	5.5	1.2
9	84.5	192.4	66.7	21.1	5.4	7.3	2.0
10	62.9	170.2	48.2	23.3	4.5	7.4	1.8
12	106.0	180.4	66.9	36.8	5.6	5.3	1.3
13	75.5	169.5	56.3	25.5	4.7	5.2	1.8
14	64.6	162.4	42.0	35.0	4.6	4.9	1.4
15	91.9	180.5	63.4	31.0	4.8	5.6	1.0
16	80.2	186.8	63.9	20.4	5.5	4.4	1.2
17	102.0	183.4	74.9	26.6	4.6	4.8	1.5
18	100.5	174.6	72.1	28.2	5.7	3.6	1.6
21	94.2	183.2	70.3	25.3	5.6	4.0	1.5
23	62.3	161.2	40.6	34.8	4.7	4.1	2.1
24	71.4	162.9	44.2	38.0	4.5	3.7	1.6
25	80.2	176.2	59.0	26.4	4.6	5.7	1.9
28	72.6	161.5	45.5	37.3	4.9	4.5	1.7
29	46.7	154.8	34.2	26.8	4.5	4.0	1.7
Female mean ± STD	66.1 ± 11.2	161.0 ± 3.1	42.2 ± 4.6	35.4 ± 4.7	4.98 ± 0.85	4.44 ± 0.65	1.60 ± 0.34
Male mean ± STD	89.8 ± 15.8	179.5 ± 7.6	65.1 ± 9.6	26.4 ± 4.6	5.16 ± 0.50	5.31 ± 1.26	1.56 ± 0.32
Overall mean ± STD	83.4 ± 18.0	174. 5 ± 10.7	58.9 ± 13.4	28.9 ± 6.1	5.11 ± 0.60	5.08 ± 1.18	1.57 ± 0.32

### Metabolic rate, ∆RQ, and surface temperatures at room temperature and cold exposure

**Fig. 1:**
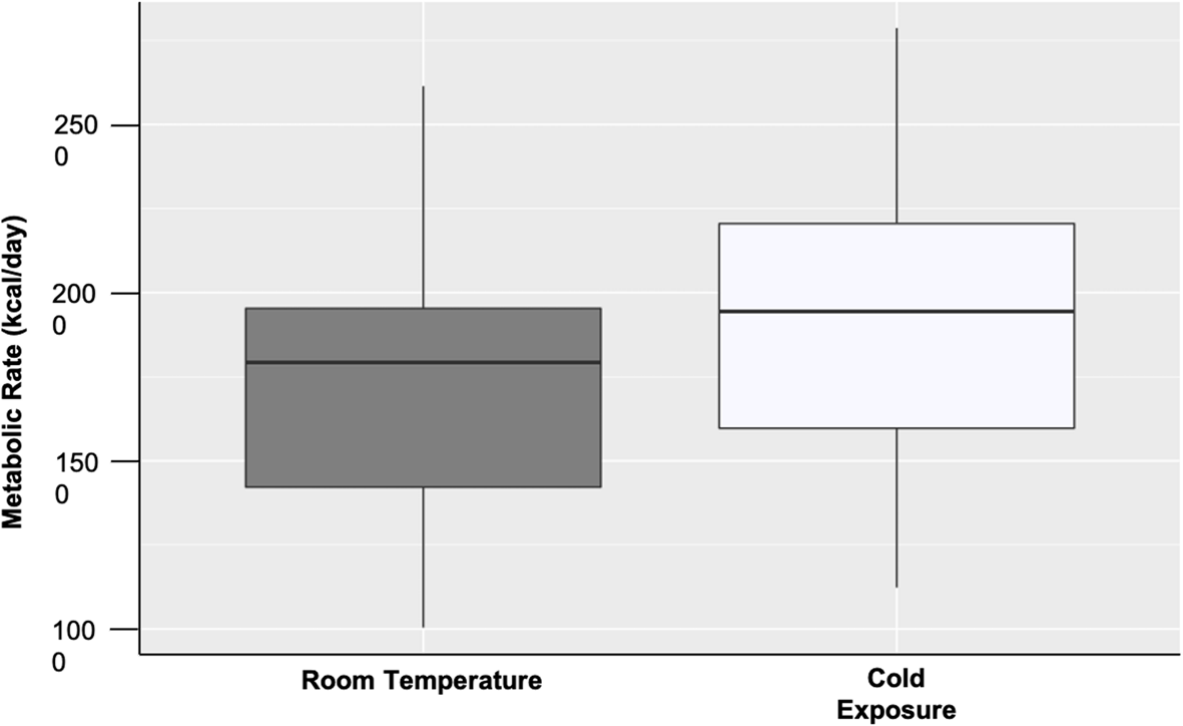
Resting metabolic rates at room temperature and mild cold exposure. Metabolic rates were overall significantly higher during cold exposure

A summary of the means ± the standard deviation for metabolic rate and surface temperatures at room temperature and during cold exposure can be found in Table 2. For a detailed description of room temperature resting metabolic rates among the reindeer herders, please see Ocobock et al. [18]. Overall, MR_RT_ ranged from 1004 to 2614 kcal/day. Among females, MR_RT_ ranged from 1599 to 2324 kcal/day. Among males, MR_RT_ ranged from 1004 to 2614 kcal/day. Females had a significantly higher MR_RT_ than males, the reasons for which has been addressed previously [18]. Overall MR_C_ ranged from 1123 to 2787 kcal/day with females ranging from 1599 to 2324 kcal/day and males ranging from 1123 to 2787 kcal/day. MR_C_ was significantly higher than MR_RT_ (*t* = − 3.629, *p* = 0.002) (Fig. 1).

**Table 2:** Metabolic rate and surface temperature data for each participant as well as the sample means for room temperature and mild cold exposure

*Participant*	*MR*_*RT*_*(kcal/day)*	*MR*_*C*_*(kcal/day)*	*SC*_*RT*_*(°C)*	*SC*_*C*_*(°C)*	*ST*_*RT*_*(°C)*	*ST*_*C*_*(°C)*	*SC*_*C*_*–ST*_*C*_*(°C)*
1	1073	1175	30.0	29.6	28.9	25.4	4.1
2	2341	2021	32.0	30.1	29.9	25.2	5.0
4	1945	2112	31.0	29.2	30.4	24.2	5.0
5	2614	2787	31.1	30.0	29.7	25.8	4.2
6	1214	1372	31.6	30.3	28.7	24.8	5.5
7	1004	1123	31.3	29.8	31.3	29.8	0.0
8	2497	2530	31.3	30.4	30.3	27.7	2.7
9	1432	1659	32.2	31.2	30.9	28.9	2.2
10	1082	1461	31.0	29.9	31.1	28.9	1.0
12	1767	1741	31.0	29.9	29.9	26.8	3.1
13	1708	1703	33.2	30.1	30.9	26.4	3.7
14	2077	2324	31.7	28.7	31.2	28.0	0.7
15	1919	2044	32.0	30.9	32.6	26.9	4.0
16	1412	1596	32.6	30.3	31.0	28.1	2.1
17	2218	2363	32.1	30.9	30.5	26.4	4.5
18	1907	2116	31.4	30.2	27.2	25.6	4.6
23	1842	1869	30.5	29.7	30.3	27.7	4.2
24	1956	2235	30.5	29.4	29.9	24.7	2.1
21	1758	2275	30.7	29.5	29.6	25.3	4.7
25	1420	1255	31.7	30.1	29.8	25.3	4.8
28	1818	2019	31.2	29.1	31.0	25.5	3.6
29	1427	1599	31.8	30.5	29.7	26.9	3.6
Female mean ± STD	1844 ± 224	2026 ± 263	31.1 ± 0.56	29.4 ± 0.62	30.4 ± 0.59	26.2 ± 1.59	3.4 ± 1.5
Male mean ± STD	1710 ± 515	1826 ± 501	31.6 ± 0.78	30.2 ± 0.47	30.1 ± 1.2	26.7 ± 1.55	3.5 ± 1.5
Overall mean ± STD	1747 ± 453	1881 ± 452	31.5 ± 0.74	30.0 ± 0.61	30.2 ± 1.1	26.6 ± 1.54	3.4 ± 1.5

**Fig. 2:**
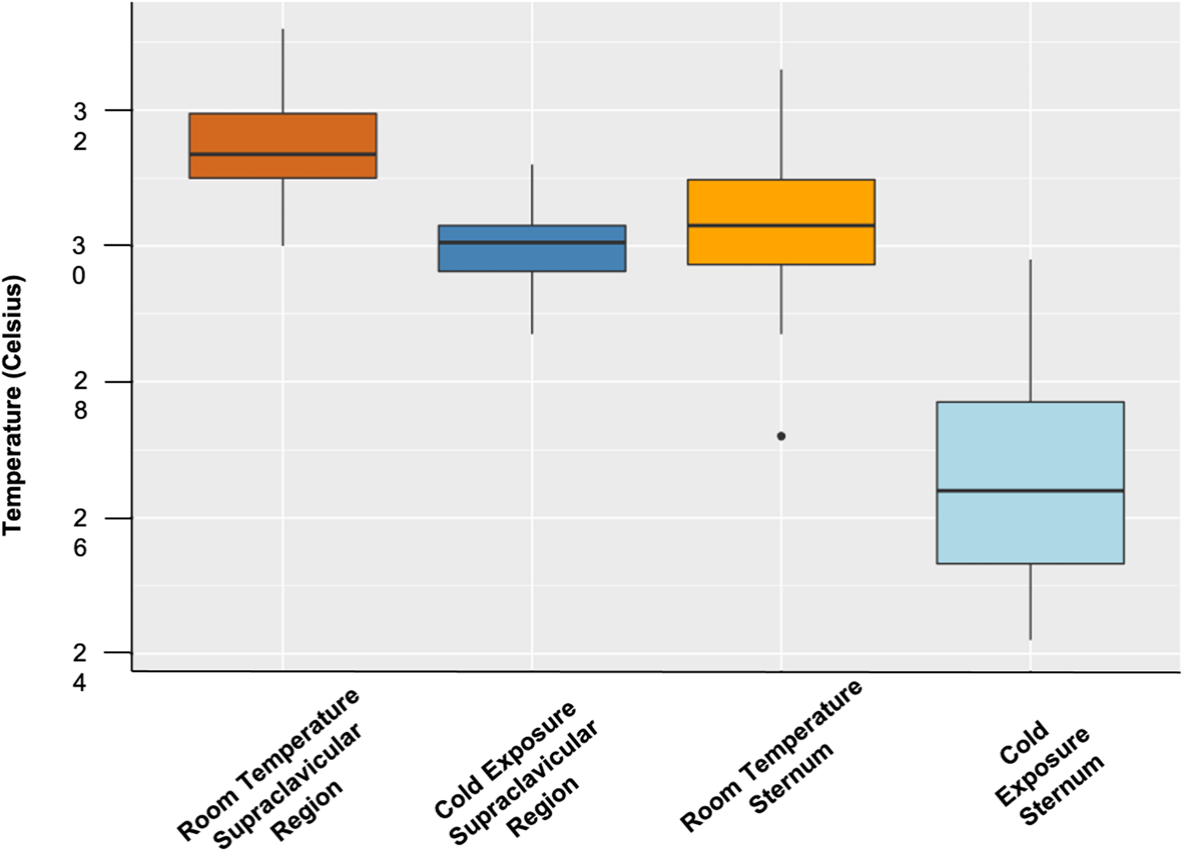
Surface temperatures at the supraclavicular region and sternum at room temperature and cold exposure. Surface temperatures significantly decreased during mild cold exposure; however, supraclavicular temperatures (known BAT location) remained significantly higher than sternum temperatures (control region without BAT)

Room temperature supraclavicular surface temperatures (SC_RT_) (Table 2) ranged from 30.0 to 33.2 °C for the overall sample and 30.5–31.8 °C among females and 30.0–33.2 °C among males. Room temperature sternum surface temperatures (ST_RT_) ranged from 27.2 to 32.6 °C for the overall sample and 29.7–31.2 °C among females and 27.2–32.6 °C among males. Cold exposure supraclavicular surface temperatures (SC_C_) ranged from 28.7 to 31.2 °C for the overall sample and 28.7–30.5 °C among females and 29.5–31.2 °C among males. Cold exposure sternum surface temperatures (ST_C_) ranged from 24.2 to 29.8 °C for the overall sample and 24.2–28.0 °C among females and 24.8–29.8 °C among males. There was no significant difference in surface temperatures between females and males for SC_RT_ (*F* = 1.723, *p* = 0.204), ST_RT_ (*F* = 0.259, *p* = 0.616), or ST_C_ (*F* = 0.522, *p* = 0.478). However, males had significantly warmer ST_C_ surface temperatures than females (*F* = 9.840, *p* = 0.005). Supraclavicular surface temperatures were significantly higher than sternum surface temperatures for both the room temperature condition (*t* = 5.274, *p* < 0.001) and cold condition (*t* = 10.675, *p* < 0.001). Room temperature surface temperatures were significantly higher than cold exposure temperatures (supraclavicular: *t* = 10.179, *p* < 0.001; sternum: *t* = 12.845, *p* < 0.001). SC_C_–ST_C_ ranged from 0 to 5.5 °C for the entire sample, 0.7–5.0 °C for females, and 0–5.5 °C for males (Table 2, Fig. 2). There was no significant difference in SC_C_–ST_C_ between males and females (*F* = 0.071, *p* = 0.792).

The mean difference ± the standard deviation in metabolic rate (∆MR), RQ (∆RQ), supraclavicular surface temperatures (∆T_SC_), and sternum surface temperatures (∆T_ST_) are listed in Table 3. Overall, ∆MR ranged from − 320 to 517 kcal/day with females ranging from 27 to 279 kcal/day and males ranging from − 320 to 517 kcal/day. For ∆RQ, the overall sample ranged from − 0.07 to 0.13 with females ranging from − 0.04 to 0.13 and males ranging from − 0.07 to 0.07. For ∆T_SC_, the overall sample ranged from − 3.10 to − 0.40 °C, females ranged from − 3.0 to − 0.80 °C, and males from − 3.1 to − 0.4 °C. For ∆T_ST_, the overall sample raged from − 6.20 to − 1.50 °C, females ranged from − 6.2 to − 2.60 °C, and males from − 5.7 to − 1.5 °C. For the whole sample, there was a significantly greater decrease in sternum surface temperatures relative to supraclavicular surface temperatures (*t* = 7.671, *p* < 0.001). There were no significant differences between the sexes for ∆MR (*F* = 0.627, *p* = 0.438), ∆RQ (*F* = 2.262, *p* = 0.148), ∆T_SC_ (*F* = 0.914, *p* = 0.350), or ∆T_ST_ (*F* = 0.1.665, *p* = 0.212).

**Table 3:** Mean metabolic rate, RQ, and surface temperature changes and percent changes associated with mild cold exposure for females, males, and the sample overall

	*Female*	*Male*	*Overall*
∆*Metabolic rate (kcal/day)*	182.2 ± 87.6	115.9 ± 195.3	134.0 ± 173.2
∆*RQ*	0.03 ± 0.06	0.001 ± 0.04	0.01 ± 0.05
∆*T*_*SC*_*(°C)*	− 1.7 ± 0.8	− 1.4 ± 0.6	− 1.5 ± 0.7
∆*T*_*ST*_*(°C)*	− 4.3 ± 1.6	− 3.4 ± 1.2	− 3.7 ± 1.3
*% Change MR*	9.9 ± 4.5	8.3 ± 12.6	8.7 ± 10.9
*% Change RQ*	5.4 ± 2.5	0.44 ± 5.3	1.78 ± 6.9
*% Change T*_*SC*_	− 5.4 ± 2.5	− 4.3 ± 1.9	− 4.6 ± 2.1
*% Change T*_*ST*_	− 14.0 ± 5.1	− 11.4 ± 3.9	− 12.1 ± 4.3

### The relationship between changes in metabolic rate, RQ, surface temperatures, and anthropometric variables

**Fig. 3:**
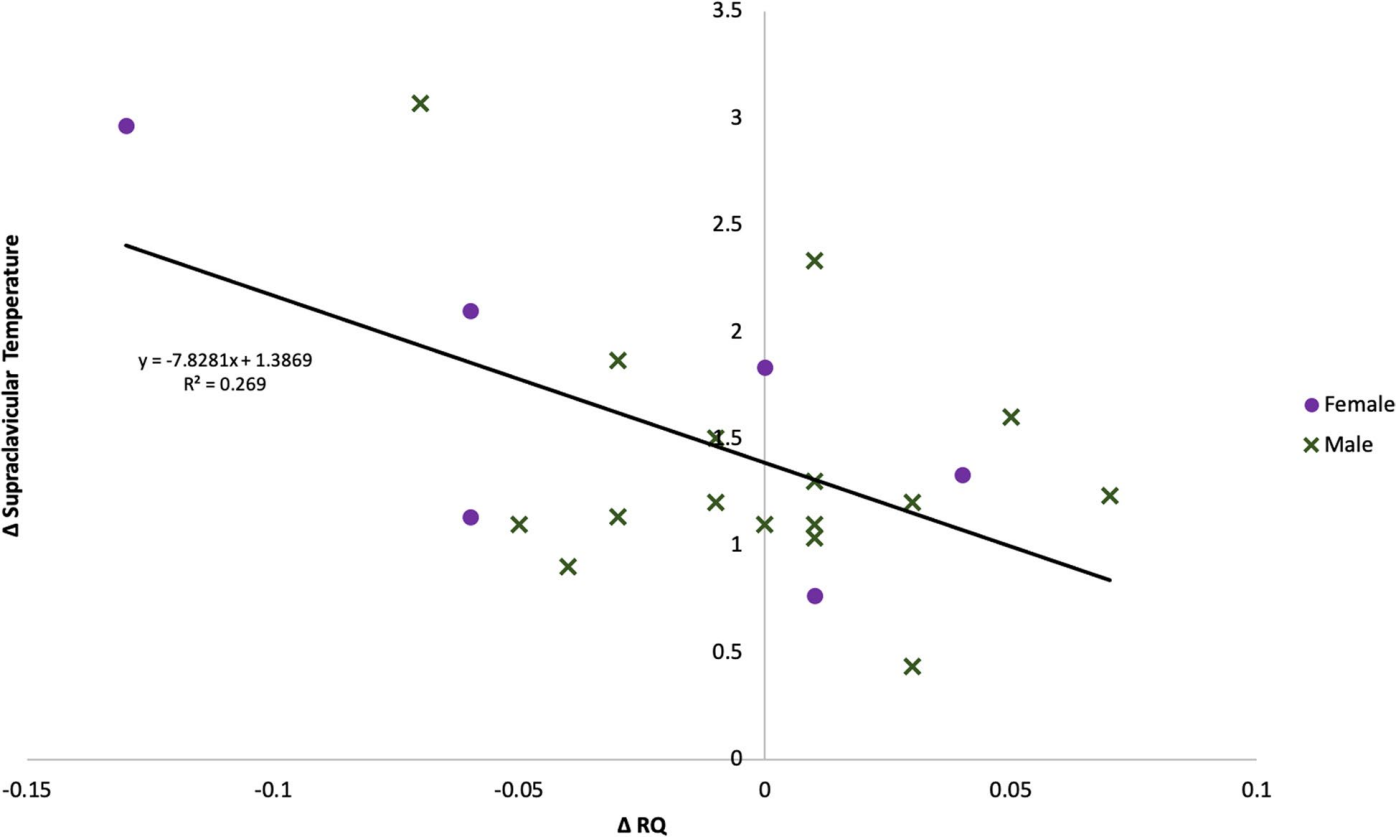
The significant negative correlation between the change in RQ and the change in supraclavicular surface temperature going from room temperature to cold exposure.

Table 4 displays a summary of the multiple linear regression results for MR_C_, ∆MR, ∆RQ, ∆T_SC_, ∆T_ST_, and SC_C_–ST_C_ assessing potential associations with age, sex, height, body mass, body fat percentage, FFM, and biomarkers. No significant regression equation was found for MR_C_ or ∆MR, ∆RQ, or ∆T_ST_. For ∆T_SC_, the only significant predictor was ∆RQ. Once the non-significant predictors were removed, the final model statistics were *R*^2^ = 0.304, *F* = 8.725, *p* = 0.008, and *β* = − 0.038 (Fig. 3). For SC_C_–ST_C_, fat free mass was a significant predictor. Once the non-significant predictors were removed, the final model statistics were *R*^2^ = 0.527, *F* = 12.711, *p* < 0.01, and *β*=1.516. None of the biomarkers were significant predictors for any regression analysis.

**Table 4:** This table presents the compiled Beta coefficient results of regression analyses for cold exposure metabolic rate, change in metabolic rate, change in RQ, change in supraclavicular and sternal surface temperatures, and the difference between supraclavicular and sternal temperatures during cold exposure. Bolded results indicate significance at *p* < 0.05. No significant predictors were found for cold exposure metabolic rate, change in metabolic rate, change in RQ, or change in sternal surface temperatures. ∆RQ was found to be a significant predictor for ∆T_SC_; final model statistics once all variables, but ∆*T*_*SC*_ were removed: *R*^2^ = 0.304, *F* = 8.725, *p* = 0.008, *β*= − 0.038. Height and fat free mass were found to be significant predictors for *SC*_*C*_*–ST*_*C*_; final model statistics once all variables, but height and fat free mass were removed: *R*^2^ = 0.527, *F* = 12.711, *p* < 0.01, *β* = 1.516. Though the results are unreported, no association was found between any of the blood biomarkers and MR_C_, ∆MR, ∆RQ, ∆T_SC_, ∆T_ST_, or SC_C_–ST_C_

	*MR*_*C*_*(kcal/day)*	∆*MR (kcal/day)*	∆*RQ*	∆*T*_*SC*_*(°C)*	∆*T*_*ST*_*(°C)*	*SC*_*C*_*–ST*_*C*_*(°C)*
	*Adj. R* ^2^ *= 0.344; F = 2.377; p = 0.080*	*Adj. R* ^2^ *= − 0.139; F = 0.679; p = 0.703*	*Adj. R* ^2^ *= − 0.227; F = 0.515; p = 0.825*	*Adj. R* ^2^ *= 0.308; F = 2.037; p = 0.125*	*Adj. R* ^2^ *= 0.015; F = 1.037; p = 0.466*	*Adj. R* ^2^ *= 0.015; F = 1.037; p = 0.466*
*Sex*	− 0.493	− 1.477	0.119	− 1.493	2.824	− 0.466
*Height (cm)*	− 0.82	0.176	− 0.43	0.954	− 0.122	− 0.533
*Mass (kg)*	0.034	0.193	− 0.434	− 0.374	0.108	− 1.455
*Age (years)*	0.064	0.479	− 0.161	0.756	− 0.044	0.107
*Body fat percentage*	1.152	− 0.346	− 0.492	− 0.962	− 0.072	0.549
*Fat free mass (kg)*	2.561	0.711	0.667	− 0.718	− 0.04	**2.471**
*Body mass index (kg/m*^*2*^*)*	− 1.204	− 0.286	0.244	0.857	− 0.353	0.216
*SC*_*C*_*–ST*_*C*_*(°C)*	− 0.319	− 0.344	− 0.022	–	–	–
∆*RQ (kcal/day)*	–	–	–	**− 0.632**	1.53	0.29
∆*MR*	–	–	0.119	− 0.351	0.001	− 0.16

## Discussion

We found that in response to mild cold exposure, herders experienced a significant increase in metabolic rate; however, there was a high degree of interindividual variation with percent change in metabolic rate ranging from − 13.7% to + 35.0%, with four of the 16 males experiencing a metabolic rate decrease. In response to cold exposure, surface temperatures decreased at both the supraclavicular region and sternum; however, the temperature decrease at the sternum was significantly greater than the supraclavicular region. The relatively warmer supraclavicular surface temperatures are indicative of BAT thermogenesis at this region as well as potentially greater heat loss through conduction with the cooling suit at the sternum. From the RQ measurements, there appeared to be no change in substrate utilization, with herders exhibiting a metabolic preference for metabolizing fatty acids in both room temperature and cold conditions. Herders did tend to have a lower RQ in both conditions, which is not uncommon among people who consume a high amount of dietary fat as the herders do [49]. There was no correlation of MR_C_, ∆MR, or ∆RQ with any anatomical or physiological variables. We found a negative correlation between ∆RQ and ∆T_SC_, suggesting that a decrease in RQ during cold exposure was associated with smaller surface temperature changes at the supraclavicular region, indicating that fatty acids were overwhelmingly utilized during cold exposure. We also found a positive correlation between SC_C_–ST_C_ and fat free mass, indicating that those with greater FFM experienced a blunted drop in supraclavicular temperature during cold exposure.

Similar work has been conducted among temperate climate populations; however, there is not a great deal of consensus. A study done among a population in Albany, NY, looked at seasonal variation in BAT activity and found a greater increase in metabolic rate in response to cold exposure during the summer relative to the winter, 12% and 11%, respectively [50]. In both seasons, though, glucose was the preferred metabolic substrate. Among Japanese males, the opposite was found, with a greater metabolic rate in response to cold exposure exhibited during the winter [51]. Nirengi et al. [48] demonstrated a comparatively blunted BAT response to cold exposure. The Japanese participants in this study had a mean SC_C_–ST_C_ of 2.2 °C, similar to that of the Yakut [19], whereas the herders in the present study had a much greater SC_C_–ST_C_–, this is indicative of greater BAT activity. Similarly, among a population from the Netherlands, there was a greater non-shivering thermogenic response after cold acclimation, an analogue to winter, than prior to acclimatization resulting in a higher metabolic rate associated with BAT activity [52]. We have summarized the results of the studies that have employed the thermal imaging methodology for assessing BAT in Supplemental Table 1.

Compared to other cold climate populations, there are a few similarities and numerous differences between the herders of this study and the Yakut participants from the only other BAT study among a circumpolar population [19, 53]. Among the few similarities are that males (four in the present study) more frequently experienced a decrease in metabolic rate during cold exposure, that there was no correlation between any demographic and anatomical variables with MR_C_, and that both populations exhibited a correlation between ∆RQ and ∆*T*_SC_. The decrease in metabolic rate is not an unusual phenomenon; however, we do not yet fully understand what might be driving this pattern among some individuals. The decrease could be due to vasoconstriction, the *Q*_10_ effect, and/or habituation [19, 42, 43], though more work is needed to elucidate the cause and pathway.

Conclusive anatomical correlates with BAT thermogenesis remain elusive with little to no correlation with fat free mass. Some studies demonstrate a negative correlation between body fat and BAT thermogenesis [54–56], some show a positive correlation [57], and many demonstrate no correlation including the present study [19, 58–61]. The lack of anatomical correlation may be surprising given BAT is thought to derive from muscle progenitor cells and beige adipose tissue appears to have a mix of white fat and muscle progenitors [62, 63].

However, work by Levy et al. [53] conducted among the Yakut suggests that environmental exposures to cold stress during key periods of childhood development may determine BAT thermogenic capacity later in life. Investigators explored if there was any association between measurements of adult BAT thermogenesis [19] and, using retrospective surveys and weather station data, cold exposure during gestation, infancy, early childhood, middle childhood, and adolescence. They found three significant associations with adult BAT thermogenesis: (1) a negative association with mean ambient temperature during early childhood and adolescence, (2) a positive association with the number of below − 40 °C days during early childhood, and (3) a positive association with participation in outdoor winter activities for ages five to seven and 11 to 13. This work among the Yakut suggests that BAT is developmentally plastic and that key periods of development likely shape BAT thermogenesis in adulthood. This is an intriguing suggestion and works well within established theory on environmental signals altering developmental trajectories [64–66].

There are a number of differences between these two cold climate populations. First, the reindeer herders (Table 2), especially the females, have higher resting metabolic rates relative to the Yakut sample—female Yakut MR_RT_ = 1129 ± 31 kcal/day, MR_C_ = 1094 ± 43 kcal/day; male Yakut MR_RT_ = 1575 ± 48 kcal/day, MR_C_ = 1528 ± 49 kcal/day. The Yakut sample also had overall warmer surface temperatures than our herder sample at the supraclavicular region (~ 36 °C vs. 30 °C) and sternum (~ 34 °C vs. 26.7 °C), for both females and males. Finally, the Yakut sample had much higher RQ values during room temperature and cold exposure, and there was a correlation between BAT thermogenesis and blood glucose levels, demonstrating a metabolic preference for carbohydrate utilization. While both populations had a correlation between ∆RQ and ∆T_SC_, herders demonstrated a negative correlation while the Yakut demonstrated a positive one. Two potential reasons for this difference could be a negative energy balance or the high-fat diet consumed by the herders in this study, which can lead to lower RQ values [49].

Despite these differences, both populations exhibited evidence of BAT activity, which could be a critical adaptation to their respective harsh, cold climates. Furthermore, the differences, such as the substrate utilization mentioned above, should not be entirely surprising as one would not and should not expect two different populations to arrive at an environmental adaptation through the same physiological routes. Differences in adaptive routes are seen elsewhere, such as the wide array of differing adaptations observed among the high altitude populations of Tibet, Peru, and Ethiopia [67, 68]. It is likely that cold climate adaptations are similarly variable and evolutionarily subject to differential selective pressures as well as genetic drift and gene flow [69].

Besides potential differences in evolutionary trajectories, there are other reasons why the reindeer herders in the present study may differ from the Yakut population. First, there are likely different developmental exposures to cold stress between these two populations. For example, the Yakut have their infants nap indoors, whereas in Finland there is a tradition for infants (2 weeks to 2 years old) to nap outside in temperatures ranging from − 27 °C to + 5 °C [70, 71]. This is a tradition we were able to witness first hand when socializing with one of the herding families. The mother prepared the infant by dressing him warmly, placing him in a stroller that had a blanket at the base, draping a blanket over the top of the stroller, and then taking him outside for a 2-to-3-h nap. Infants napping outdoors could have a significant impact on adult BAT thermogenesis. Furthermore, given the need for outdoor physical activity among reindeer herders throughout winter and the strong cross country skiing culture in Finland, even among very young children [72], it is likely that children in this population have a high level of cold exposure during key developmental periods. The potential for BAT developmental plasticity among the herders in the present study is an exciting avenue for future research on adaptive responses to cold stress across the life course.

Seasonal acclimatization is another possible reason for the differences seen in this population, since in the present study, BAT thermogenesis was assessed during the winter, while the study among the Yakut was performed during early fall. There is a known increase in BAT thermogenesis during the winter among people living in temperate climates [52, 58, 73]. Furthermore, there appears to be a seasonal difference in BAT substrate utilization, at least among a population in Albany, NY, in which carbohydrate utilization during BAT thermogenesis is higher in the winter than it is in the summer [50]. This indicates that BAT thermogenesis is not only plastic during key developmental stages but that it is also highly responsive to environmental cues in adulthood. Gathering more seasonal data on BAT thermogenesis among the herders and other cold climate populations will help elucidate seasonal and well as populational variation.

Finally, other confounding factors that may lead to variation in BAT thermogenesis, particularly substrate utilization, are the levels of physical activity and dietary intake during the days leading up to the measurement. The herders in this sample had an exceptionally high total energy expenditure during the annual herd roundup in the fall [49]. Though physical activity levels in the winter are likely not as high as they are in the fall, herders typically provide supplemental feed to their herds—a demanding chore [74]. At this time, herders also prefer to consume energy-rich foods. The RQ associated with BAT thermogenesis in this study could have been affected if in the days before the BAT measurements herders were highly active and consumed fat-rich foods. This situation could have left the herders, who were at least 12 h fasted at the time of measurement, with relatively depleted stores of glycogen [75] and more readily available fatty acids for BAT to utilize. Future work should account for diet and activity levels in the days prior to BAT thermogenesis assessment.

There are a number of limitations to the present study. First, there was a small number of participants reducing the overall statistical power. Second, males in this study outnumbered females. Though the sex-bias reflects the current reindeer herder demographics [44], the small number of females makes it difficult to determine if there may be any sex-based differences in BAT thermogenesis. Third, this data is cross-sectional in nature as the data collection took place only in winter. As mentioned above, BAT thermogenesis is sensitive to seasonal temperatures. To get a broader view of BAT variation in this population, seasonal measures are needed. Fourth, to better understand BAT substrate utilization, it would be beneficial to have whole blood measures of glucose and cholesterol before and after cold exposure in addition to the RQ measurement. Fifth, these measures took place in several different locations for which room conditions (temperature and humidity) could not be easily controlled. Future work should utilize one location for data collection. Finally, this study did not collect data on childhood cold stress nor physical activity and dietary intake in the days leading up to the BAT measurement. These factors likely alter BAT thermogenesis and should be included in any future studies.

## Conclusions

This study assessed BAT thermogenesis among a small sample of Finnish reindeer herders from sub-Arctic Finland. We found that the herders do indeed have active BAT thermogenesis that resulted in a mean ~8.7% increase in metabolic rate in response to a mild cold stress. There were no statistically detectable anatomical correlates with BAT thermogenesis in this sample, though that is common among other BAT studies. Finally, this sample demonstrated a metabolic preference for fatty acid utilization during both room temperature and mild cold exposure, which was different from findings among the circumpolar Yakut. This study adds to the rapidly growing body of work looking at the physiological and thermoregulatory significance of brown adipose tissue and the key role it may play among cold stressed populations. The current state of brown adipose research is one in which we have far more questions than answers, making it rich topic for future research.

## Supplementary Information

**Additional file 1.**

## Data Availability

The dataset supporting the conclusions of this article is included within the article, with the age and sex removed to protect participant confidentiality. The full dataset will be made available upon request.

## Abbreviations

BAT: Brown adipose tissue
RMR: Resting metabolic rate
TEE: Total energy expenditure
HDL: High density lipoprotein
FFM: Fat free mass
RQ: Respiratory quotient
MR: Metabolic rate
MR_RT_: Room temperature metabolic rate
MR_C_: Cold exposure metabolic rate
RQ_RT_: Room temperature respiratory quotient
RQ_C_: Cold exposure metabolic rate
T_SC_: Supraclavicular surface temperature
T_ST_: Sternum surface temperature
ST_RT_: Room temperature sternum surface temperature
ST_C_: Cold exposure sternum surface temperature
SC_RT_: Room temperature supraclavicular surface temperature
SC_C_: Cold exposure supraclavicular surface temperature
