# Supplementary material for: Brown adipose tissue thermogenesis among a small sample of reindeer herders from sub-Arctic Finland
Source: J Physiol Anthropol. 2022 Apr 20;41:17. doi: 10.1186/s40101-022-00290-4 (PMC9022279; doi:10.1186/s40101-022-00290-4)
Supplement: Supplementary file 1 — Additional file 1. [file 40101_2022_290_MOESM1_ESM.docx]

|  | Levy et al., 2018 | | Niclou and Ocobock, 2021 | | | | Nirengi et al. 2019 | Present Study | |
| --- | --- | --- | --- | --- | --- | --- | --- | --- | --- |
| Population | Yakutia, Siberia | | Albany, New York | | | | Kyoto, Japan | Lapland, Finland | |
| Participants | M: n=31 | F: n=43 | Summer  M: n=22 F: n=37 | | Winter  M: n=23 F: n=36 | | M: n=24 | M: n=16 | F: n=6 |
| Age (yrs) | 28.8 ± 8.0 | 27.4 ± 8.7 | 31.2 ± 7.0 | 26.8 ± 7.1 | 30.1 ± 8.8 | 27.7 ± 8.3 | 23.5 ± 3.6 | 50.5 ± 9.9 | 27.7 ± 6.9 |
| Body Mass (kg) | 66.2 ± 11.1 | 54.4 ± 8.9 | 92.0 ± 16.3 | 73.2 ± 16.5 | 92.5 ± 17.8 | 73.8 ± 16.2 | 64.0 ± 8.6 | 89.8 ± 15.8 | 66.1 ± 11.2 |
| BMI | 22.4 ± 3.6 | 21.6 ± 3.6 | 28.4 ± 4.1 | 26.9 ± 5.4 | 28.6 ± 3.8 | 27.0 ± 5.3 | 21.6 ± 2.5 | - | - |
| BF% | 21.4 ± 5.5 | 33.2 ± 4.6 | 24.7 ± 5.6 | 36.4 ± 8.8 | 26.0 ± 5.3 | 36.6 ± 8.1 | 16.6 ± 4.4 | 26.4 ± 4.6 | 35.4 ± 4.7 |
| FFM (kg) | 55.8 ± 5.8 | 41.0 ± 5.4 | 69.7 ± 9.3 | 45.9 ± 6.6 | 67.7 ± 9.9 | 44.9 ± 9.0 | 35.6 ± 1.8* | 65.1 ± 9.6 | 42.2 ± 4.6 |
| MR_RT_ (kcal/day) | 1575 ± 48 | 1129 ± 31 | 1905 ± 474 | 1534 ± 391 | 1919 ± 615 | 1629 ± 374 | - | 1710 ± 515 | 1844 ± 224 |
| MR_C_ (kcal/day) | 1528 ± 49 | 1094 ± 43 | 1997 ± 506 | 1793 ± 457 | 2080 ± 645 | 1857 ± 475 | - | 1826 ± 501 | 2026 ± 263 |
| *SC_RT_* (°C) | 36.8 ± 0.07 | 36.6 ± 0.07 | 31.4 ± 1.0 | 31.1 ± 1.0 | 32.8 ± 0.7 | 32.5 ± 0.7 | 33.0 ± 0.7 | 31.6 ± 0.78 | 31.1 ± 0.56 |
| *SC_C_* (°C) | 36.8 ± 0.06 | 36.6 ± 0.07 | 30.4 ± 1.0 | 30.2 ± 0.8 | 31.8 ± 0.8 | 31.2 ± 0.7 | 31.9 ± 0.5 | 30.2 ± 0.47 | 29.4 ± 0.62 |
| *ST*_RT_ (°C) | 34.8 ± 0.14 | 38.8 ± 0.13 | - | - | - | - | 33.1 ± 0.4 | 30.1 ± 1.2 | 30.4 ± 0.59 |
| *ST*_C_ (°C) | 34.3 ± 0.13 | 34.2 ± 0.14 | - | - | - | - | 29.7 ± 0.3 | 26.7 ± 1.55 | 26.2 ± 1.59 |
| *SC_C_ - ST_C_ (°C)* | 2.5 ± 1.7 | 2.4 ± 1.7 | - | - | - | - | 2.2 ± 1.6 | 3.5 ± 1.5 | 3.4 ± 1.5 |
| RQ_RT_ | 0.85 ± 0.02 | 0.8 ± 0.01 | 0.75 ± 0.06 | 0.76 ± 0.05 | 0.80 ± 0.05 | 0.79 ± 0.04 | - | 0.69 ± 0.05 | 0.67 ± 0.03 |
| RQ_C_ | 0.86 ± 0.02 | 0.84 ± 0.02 | 0.74 ± 0.08 | 0.76 ± 0.07 | 0.82 ± 0.09 | 0.78 ± 0.05 | - | 0.69 ± 0.04 | 0.70 ± 0.04 |

Supplemental Table 1: Summary of brown adipose positive results that utilize thermal imaging to infer brown adipose tissue heat dissipation. The * indicates where the data collected was skeletal muscle mass rather than fat free mass as was typical in all other studies.
